# Transcriptome-Wide Association Study Reveals New Molecular Interactions Associated with Melanoma Pathogenesis

**DOI:** 10.3390/cancers16142517

**Published:** 2024-07-11

**Authors:** Mohamed N. Saad, Mohamed Hamed

**Affiliations:** 1 Biomedical Engineering Department, Faculty of Engineering, Minia University, Minia 61519, Egypt; 2 Institute for Biostatistics and Informatics in Medicine and Ageing Research (IBIMA), Rostock University Medical Center, 18057 Rostock, Germany; mohamed.hamed@uni-rostock.de; 3 Faculty of Media Engineering and Technology, German University in Cairo, Cairo 11835, Egypt

**Keywords:** gene expressions, genome-wide association study summary statistics, melanoma, molecular interactions, transcriptome-wide association study

## Abstract

**Simple Summary:**

The journey of discovering melanoma—the most dangerous type of skin cancer—biomarkers is never-ending. Under that assumption, this study attempts to partially fill a gap in that journey by identifying melanoma-related biomarkers. This research paper studied the genetic and molecular profiling of melanoma. A transcriptome-wide association study (TWAS) was conducted to reveal the significant biomarkers associated with melanoma. A molecular network was constructed to represent the significant interactions between genes and microRNAs (miRNAs). The related biological processes, the enriched pathways, and the associated diseases were identified. The hotspot biomarkers were discovered to elucidate further the mechanism of these genes and miRNAs in melanoma pathogenesis. This work reveals the pathogenesis and biologically important molecular interactions of melanoma and provides new insights into the molecular mechanisms of melanoma.

**Abstract:**

A transcriptome-wide association study (TWAS) was conducted on genome-wide association study (GWAS) summary statistics of malignant melanoma of skin (UK Biobank dataset) and The Cancer Genome Atlas-Skin Cutaneous Melanoma (TCGA-SKCM) gene expression weights to identify melanoma susceptibility genes. The GWAS included 2465 cases and 449,799 controls, while the gene expression testing was conducted on 103 cases. Afterward, a gene enrichment analysis was applied to identify significant TWAS associations. The melanoma’s gene–microRNA (miRNA) regulatory network was constructed from the TWAS genes and their corresponding miRNAs. At last, a disease enrichment analysis was conducted on the corresponding miRNAs. The TWAS detected 27 genes associated with melanoma with *p*-values less than 0.05 (the top three genes are *LOC389458* (*RBAK*), *C16orf73* (*MEIOB*), and *EIF3CL*). After the joint/conditional test, one gene (*AMIGO1*) was dropped, resulting in 26 significant genes. The Gene Ontology (GO) biological process associated the extended gene set (76 genes) with protein K11-linked ubiquitination and regulation of cell cycle phase transition. K11-linked ubiquitin chains regulate cell division. Interestingly, the extended gene set was related to different skin cancer subtypes. Moreover, the enriched pathways were nsp1 from SARS-CoV-2 that inhibit translation initiation in the host cell, cell cycle, translation factors, and DNA repair pathways full network. The gene-miRNA regulatory network identified 10 hotspot genes with the top three: *TP53*, *BRCA1*, and *MDM2*; and four hotspot miRNAs: mir-16, mir-15a, mir-125b, and mir-146a. Melanoma was among the top ten diseases associated with the corresponding (106) miRNAs. Our results shed light on melanoma pathogenesis and biologically significant molecular interactions.

## 1. Introduction

Genome-wide association studies (GWASs) aim to investigate the relationship between a genotype and a phenotype through a group of cases and controls [1]. Through the past two decades, GWAS has successfully identified many single nucleotide polymorphisms (SNPs) statistically associated with monogenic and complex diseases [2,3]. However, GWASs lack the causative biological meaning of their identified variants. Moreover, the linkage disequilibrium (LD) among SNPs overshadows the causative variants [4].

The statistically significant variants (biomarkers) identified from a well-conducted GWAS followed by an LD study could be as follows: (1) coding variants: affecting the sequence of specific genes by either stop/loss, stop/gain, frameshift, missense mutations, or large deletions; or (2) regulatory variants: affecting gene switches or participating in posttranscriptional gene regulation. For most complex diseases, most of the biomarkers (90%) appear to be regulatory variants; however, coding variants are the exception [5].

To shed light on the GWAS results, a fine mapping should be conducted to identify the causal genes leading to the biological mechanisms and the downstream pathways contributing to the disease. One of the most widespread fine-mapping techniques is the transcriptome-wide association study (TWAS). In the case of regulatory variants, it is hard to identify the causal gene as they change the expression levels of many target genes. TWAS aims to integrate the GWAS results with the gene expression datasets to prioritize the GWAS-associated variants—mostly regulatory variants—and detect the causal genes [6].

Melanoma is a typical form of cancer that mainly affects the skin, iris, and rectum. While melanoma represents 4% of skin cancers, it is responsible for 75% of deaths due to this type of cancer. Australian and New Zealand populations are affected by melanoma at a rate of approximately 0.06% per year. The disease is also spread in Europe and northern America, mainly among White populations. In Africans and Asians with dark skin, the spread of the disease is as low as one patient per 100,000 per year. It most often starts within 40–60 years of age. Women’s survival rate is better than men’s for no known reason.

It is believed that the tendency to develop melanoma may be genetically inherited. Moreover, environmental factors, such as dense ultraviolet (UV) exposure, may excite and develop the disease in genetically susceptible children—or adults—mainly through DNA damage. Melanoma could be diagnosed by the naked eye, dermoscopy, biopsy of benign skin lesions, sequential digital dermoscopic imaging, in vivo reflectance confocal laser microscopy, computer-aided multispectral digital analysis, or electrical impedance spectroscopy [7,8]. The brain, lungs, liver, and bones are target sites for melanoma metastasis [9].

Meta-analysis GWAS have marked 54 loci that may be associated with cutaneous melanoma susceptibility. This GWAS suggested nevus formation, pigmentation, and telomere maintenance as biological pathways contributing to cutaneous melanoma [10]. Five genes (*ZFP90*, *HEBP1*, *MSC*, *CBWD1*, and *RP11-383H13.1*) were detected as melanoma biomarkers by TWAS using melanocyte eQTLs (expression quantitative trait loci) [11]. Fidalgo et al. identified *MYO7A*, *WRN*, *SERPINB4*, *HRNR*, and *NOP10* through whole exome sequencing (WGS) as rare variants for cutaneous melanoma in Brazilian families [12].

The top 10 genes, *EGFR*, *IL8*, *ICAM1*, *STAT1*, *CDK2*, *OAS2*, *MITF*, *PBK*, *CDKN3*, and *CXCL10*, were confirmed for association with melanoma through bioinformatic analysis in differentially expressed genes (DEGs) [13]. Logit regression and survival analysis were applied to detect the eleven feature DEGs, *ZNF750*, *NLRP6*, *TGM3*, *KRTDAP*, *CAMSAP3*, *KRT6C*, *CALML5*, *SPRR2E*, *CD3G*, *RTP5*, and *FAM83C*, related to the prognosis of metastatic cutaneous melanoma [14].

*MDM2*, *MDM4*, *USP7*, and *PPM1D* were confirmed in metastatic melanoma patients [15]. Spanish individuals bearing wildtype *MC1R* were predisposed to melanoma with a main role of the *HERC2* gene. *ESR1* was protective against naevus count in female melanoma patients [16]. Walbrecq et al. suggested six proteins (AKR7A2, DDX39B, EIF3C, FARSA, PRMT5, and VARS) and four microRNAs (miRNAs) (miR-210, miR-1290, miR-23a-5p, and miR-23b-5p) as biomarkers for melanoma [17].

**Figure 1:**
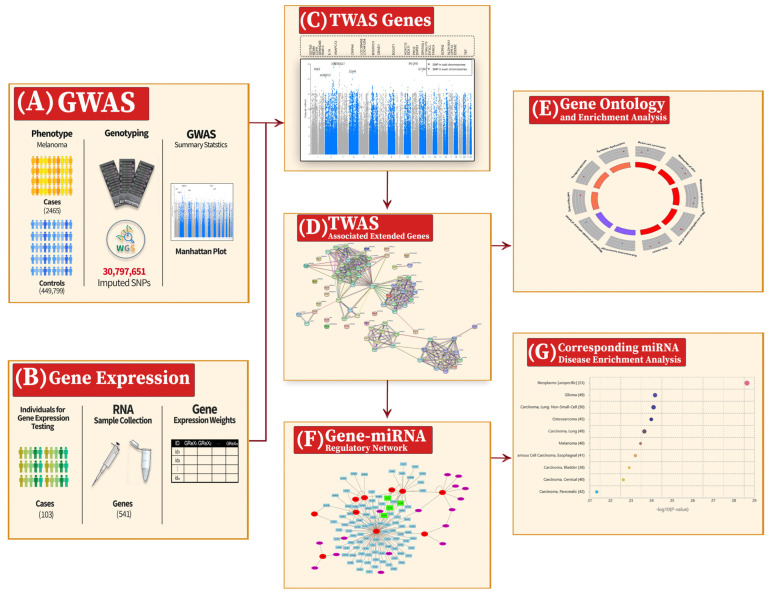
Summary of the proposed system for the transcriptome-wide association study (TWAS) and the extended analysis. (**A**) 2465 melanoma patients and 449,799 healthy individuals were genotyped for 30,797,651 SNPs after imputation, resulting in the genome-wide association study (GWAS) summary statistics. (**B**) The expression weights for 541 genes were calculated for 103 melanoma patients. (**C**) The lower part represents the Manhattan plot for the GWAS; the upper part shows the associated genes—resulting from the TWAS—at their corresponding chromosomal positions. (**D**) The expanded network of 76 genes from the 26 TWAS-associated genes using protein–protein interactions to enhance the relevance of the functional analysis. (**E**) A gene ontology (GO) and enrichment analysis for the 76 genes. (**F**) The constructed gene–miRNA regulatory network resulting in 106 corresponding miRNAs to the extended gene set. (**G**) A disease enrichment analysis for the 106 miRNAs showing melanoma in the top ten diseases.

This study aims to conduct a TWAS to identify the causal genes of cutaneous melanoma using online available GWAS summary statistics and a gene expression dataset. After extending the causal genes by protein–protein interaction network, they will be related to melanoma pathology and linked to other molecular interactions and gene–disease associations by applying a functional pathway analysis and disease enrichment analysis. Moreover, a gene–miRNA regulatory network will be constructed to elucidate further the mechanism of the extended genes in melanoma pathogenesis. Furthermore, the corresponding miRNAs’ disease enrichment analysis will be conducted to validate their association with melanoma. Figure 1 shows a block diagram of the entire association study.

## 2. Materials and Methods

### 2.1. GWAS Summary Statistics Dataset for Melanoma

The UK Biobank is a nonprofit project considered an open resource for big medical datasets. It includes datasets of various categories, such as clinical data, demographic data, biomedical images, and genetic data. The genetic data were collected from about 500,000 participants, and 90 million SNPs, after imputation, were made available in July 2017 [18].

The Gene Atlas is a massive database for UK Biobank cohort associations, including 452,264 individuals, 778 traits, and over 30 million genetic variants [19]. The malignant melanoma of skin (C43) trait is selected from the Gene Atlas as the GWAS summary statistics dataset for the TWAS analysis (http://geneatlas.roslin.ed.ac.uk/trait/?traits=60, accessed on 16 July 2021). It consists of 2465 cases and 449,799 controls, with 30,797,651 imputed SNPs (after excluding the X chromosome). The participating individuals are from both sexes and have White British ancestry.

### 2.2. Gene Expression Dataset for Melanoma

The Cancer Genome Atlas (TCGA) data portal is a systematic cancer genomics project to identify new cancer biomarkers. The tumors included in the project are studied on different levels, such as DNA methylation, epigenetics, mRNA and miRNA expressions, copy number variation, the genome, and the whole exome [20]. The TCGA-SKCM (Skin Cutaneous Melanoma) is the gene expression dataset with 103 samples of White ancestor origin to match the ethnic group of the GWAS samples and 541 features whose expression profiles are significantly dysregulated compared to normal samples.

### 2.3. Transcriptome-Wide Association Study (TWAS)

The Functional Summary-based Imputation (FUSION) is a pipeline R package used to conduct a TWAS. It identifies genetic biomarkers by integrating SNPs, gene expressions, and trait status. The strength of FUSION is the ability to perform a TWAS with GWAS summary statistics only—that are likely to be found freely online—without the need to apply the gene expression study on the same individuals [21].

First, the melanoma GWAS summary statistics dataset was preprocessed to be appropriate for FUSION tool analysis by computing Z-scores equivalent to SNPs’ *p*-values using p.to.Z in R (https://www.R-project.org/, accessed on 15 August 2021). Second, the precomputed gene expression weights for TCGA-SKCM were downloaded from the FUSION website (http://gusevlab.org/projects/fusion/, accessed on 25 January 2022). Finally, the imputation methods for gene expression in genotyped samples were cis-eQTL, least absolute shrinkage and selection operator (LASSO), and elastic net [22].

For each gene, the GWAS SNPs within the range of 500 kbp from the gene locus in both directions were selected for testing. The statistically significant SNP detected by GWAS could be associated with more than one gene regulating their expressions.

The TWAS-associated gene set was searched for other diseases’ associations using ClinVar (https://www.ncbi.nlm.nih.gov/clinvar/, accessed on 22 March 2022) [23]. Only diseases confirmed by at least one PubMed publication by ClinVar were included. A Manhattan plot integrating the associated TWAS genes was implemented using LocusZoom.js (https://my.locuszoom.org/, accessed on 11 December 2021) [24].

### 2.4. Statistical Test

Statistical analysis of TWAS was performed using the moderated t-test followed by false discovery rate (FDR) correction, Bonferroni correction, or permutation testing. The TWAS *p*-values less than 0.05 were considered statistically significant.

### 2.5. Expansion of TWAS-Associated Genes with Partner Proteins

The STRING is a database for protein–protein physical and functional interactions. It covers all the publicly available resources and integrates them into one repository [25]. After applying the TWAS, the STRING (https://string-db.org/, accessed on 12 April 2022) database was used to extend the associated gene set by other interacting proteins. The maximum number of interactors in the first shell was set to fifty.

### 2.6. Gene Ontology (GO) and Enrichment Analysis

The Enrichr is a comprehensive gene set enrichment analysis tool [26,27,28]. The extended gene set, constructed by STRING, was operated by Enrichr (https://maayanlab.cloud/Enrichr/, accessed on 18 June 2022), and the results of GO Biological Process 2021, WikiPathway 2021 Human, and PheWeb 2019 databases were obtained.

The GOplot is an R package for visualizing and interpreting gene expression data with enrichment analysis [29]. The bubble plot was used to visualize the results of the GO Biological Process 2021 and WikiPathway 2021 Human databases. The circle plot was applied to display the relationship between the TWAS genes and the selected diseases resulting from PheWeb 2019.

The fold change (FC) for each gene was calculated using the GEPIA2 (Gene Expression Profiling Interactive Analysis version 2) tool (http://gepia2.cancer-pku.cn/#index, accessed on 12 November 2022), which is an essential preparation step for the GOplot package to produce the bubble and circle plots [30,31]. The GeneCards database (https://www.genecards.org, accessed on 30 October 2022) was used to find the aliases for the extended gene set for further analysis [32].

### 2.7. Corresponding MicroRNA and Disease Enrichment Analysis

TFmiR2 is a web server for analyzing and constructing transcription factor (TF) and miRNA coregulatory networks [33]. For further analysis, TFmiR2 was utilized to obtain the corresponding miRNA set for the extended gene set in response to melanoma disease. The gene–miRNA regulatory network was visualized using the Cytoscape 3.8.2 tool [34]. Hence, the miRNA set was exposed to TAM 2.0 for disease enrichment analysis [35].

## 3. Results

A TWAS was conducted on melanoma GWAS summary statistics and TCGA-SKCM gene expression weights. Table 1 includes the significant associations of the TWAS. Moreover, Table 1 included the previous studies for the identified genes linked to melanoma, if any, along with association with other diseases explored from the ClinVar archive, if any. The FC for each gene was calculated from the GEPIA 2 website for SKCM tumors and normal individuals. The results showed that the expressions of twenty-seven genes were significantly associated with melanoma GWAS SNPs with TWAS *p*-values less than 0.05. No associations were detected for the five chromosomes (5, 6, 10, 18, and 21).

**Figure 2:**
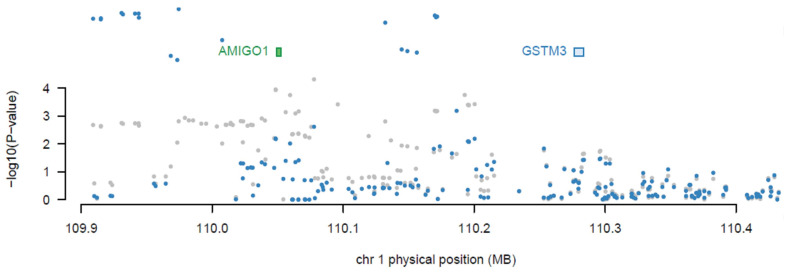
Joint/conditional test result for melanoma TWAS chromosome 1 significant genes. The TWAS-associated genes are highlighted in blue, and those jointly significant are highlighted in green. The bottom panel shows a Manhattan plot of the GWAS data before (gray) and after (blue) conditioning on the green genes, and the *AMIGO1* gene was dropped out.

**Figure 3:**
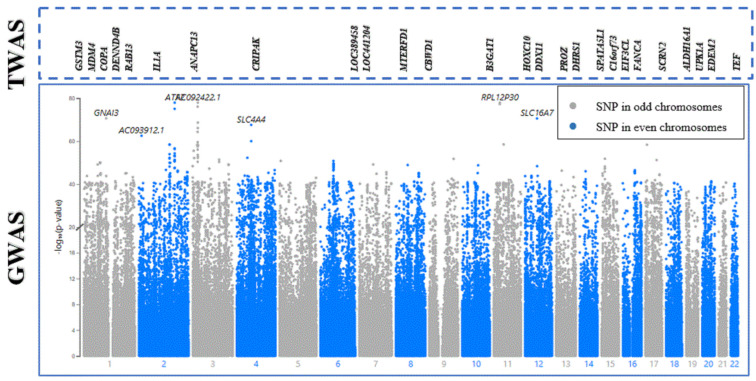
Melanoma TWAS-significant genes integrated with a Manhattan plot using melanoma GWAS summary statistics from the Gene Atlas database. The X-axis plots chromosome number. The gray and blue colors in the Manhattan plot represent SNPs in alternating chromosomes. The Y-axis shows −log10(*p*-values), representing the strength of association between the SNP and melanoma. The higher the SNP (point) on the scale, the more significant the association with melanoma susceptibility. For the TWAS section, the significantly expressed genes are ordered according to their physical positions and designated above their corresponding chromosome number in the GWAS section.

Subsequently, joint/conditional tests were applied to detect any conditionally dependent genes in the same chromosomic region. The chromosomes that had more than one significant gene were tested—the tested chromosomes were 1, 7, 12, 16, and 19. The gene (*AMIGO1*) in chromosome 1 was detected to be dependent on another gene (*GSTM3*) with a joint *p*-value of 0.0059, as shown in Figure 2. Then, *AMIGO1* (conditionally nonsignificant gene) was dropped from the list of the associated genes. Consequently, the upcoming analysis was applied to 26 significant genes only. Figure 3 represents the significantly expressed 26 genes resulting from FUSION TWAS, combined with the LocusZoom plot for GWAS summary statistics.

## 4. Discussion

**Figure 4:**
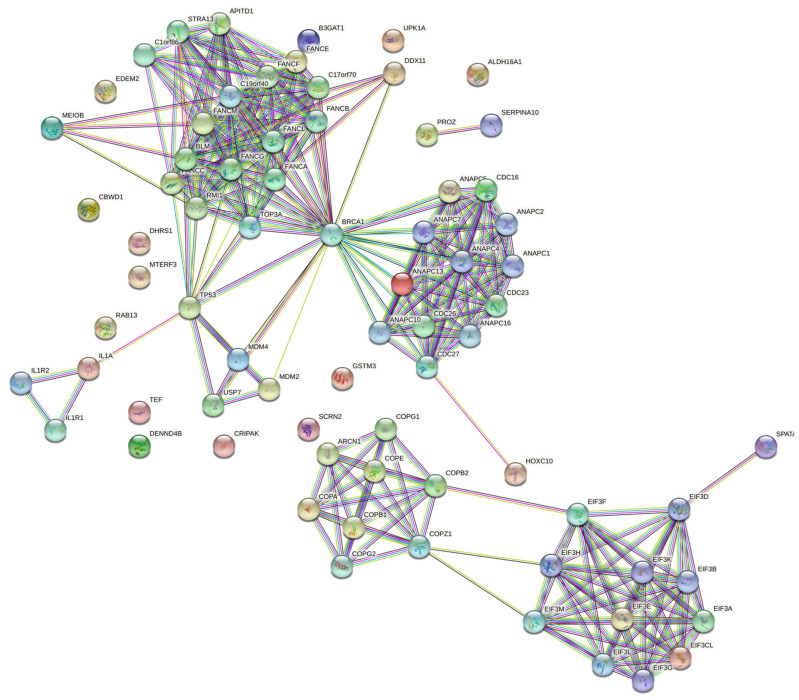
The TWAS-expressed genes were extended from 26 to 76 using STRING protein–protein interaction networks.

To reveal the associated genes with melanoma, a TWAS was performed using malignant melanoma of skin (C43) GWAS summary statistics (30,797,651 imputed SNPs with 2465 cases and 449,799 controls) and TCGA-SKCM precomputed gene expression weights (103 samples and 541 features). In total, 27 genes were considered promising candidates for melanoma, and a dependent gene was recognized through joint/conditional tests. To apply the enrichment analysis on a proper number of genes, the corresponding 26 proteins were networked to their physically and functionally partner proteins, resulting in 76 proteins using STRING, as shown in Figure 4. Table 2 lists the extended gene set with their corresponding Ensembl IDs (from GEPIA 2 website) including aliases (from GeneCards database), if any.

**Figure 5:**
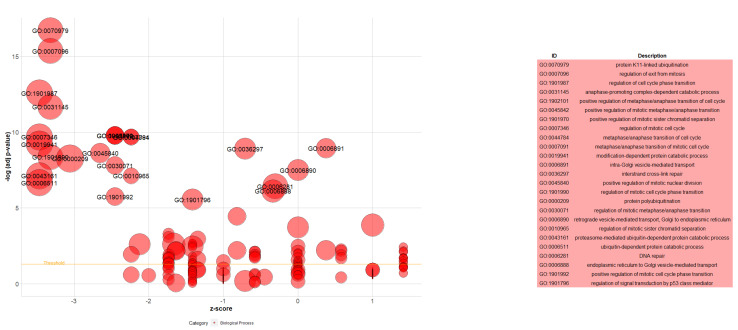
Bubble plot of the enriched GO terms in combination with the extended genes set. Each bubble represents a GO term. The Y-axis shows −log10 (adjusted *p*-value) representing the strength of association between the GO term and the extended genes set. The X-axis demonstrates the Z score (the number of assigned genes downregulated (logFC < 0) subtracted from the number of assigned genes upregulated (logFC > 0) to the GO term divided by the square root of the number of genes assigned to the GO term). The bubble’s position on the plot corresponds to the GO term’s significance (the higher, the more significant). The size of each bubble represents the number of genes associated with the GO term. The bubbles are labeled with the GO term identifiers (IDs). A table displays the IDs, and the corresponding term description can be found on the right side.

The 76 genes, detected by FUSION TWAS and extended by STRING, were further functionally analyzed by conducting GO and enrichment analysis using Enrichr. GO and enrichment analyses were carried out on the expressed genes based on GO Biological Process 2021, WikiPathway 2021 Human, and PheWeb 2019 databases. For the GO Biological Process 2021 database, the extended differentially expressed genes were involved in protein K11-linked ubiquitination, regulation of exit from mitosis, regulation of cell cycle phase transition, anaphase-promoting complex-dependent catabolic process, and more (visualized by GOplot package in Figure 5).

**Figure 6:**
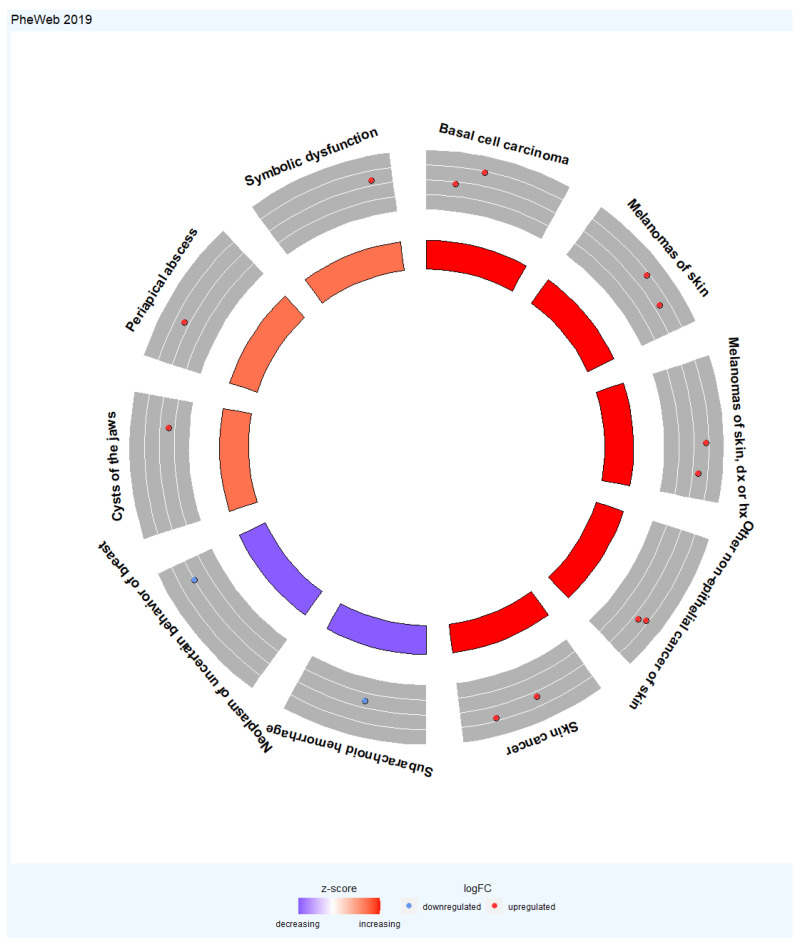
Circular visualization of the enriched phenotypes with the extended TWAS genes set. The outer circle represents the top ten enriched phenotypes. The middle circle shows a scatter plot for each phenotype of the logFC of the assigned genes. The red circles display upregulation, and the blue ones display downregulation concerning logFC. The inner circle represents the Z score for the genes associated with these phenotypes. The Z score heatmap is used to describe the expression levels of genes. The denser red color represents a higher Z score value, and the denser blue color represents a lower Z score value. In some cases, highly significant phenotypes have a Z score value close to zero with white color.

For the PheWeb 2019 database, the extended TWAS-identified genes were associated with basal cell carcinoma, melanomas of skin, melanomas of skin, dx or hx, other non-epithelial cancer of skin, skin cancer, subarachnoid hemorrhage, neoplasm of uncertain behavior of breast, cysts of the jaws, periapical abscess, and symbolic dysfunction (Figure 6).

**Figure 7:**
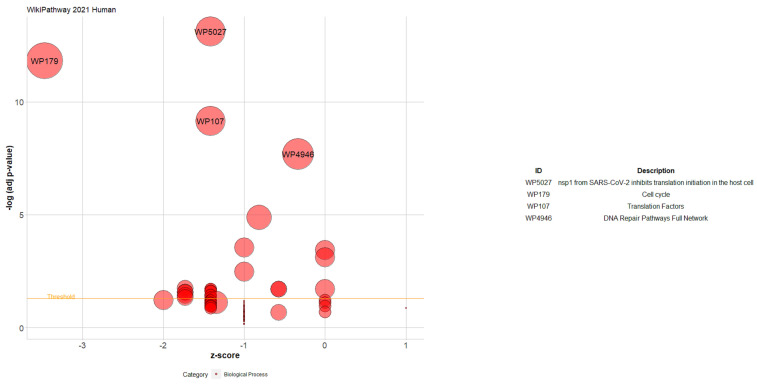
Bubble plot of the enriched pathways related to the extended TWAS genes. Each bubble represents a pathway. The Y-axis shows −log10 (adjusted *p*-value), representing the strength of association between the pathway and the extended TWAS genes. The X-axis demonstrates the Z score (the number of assigned genes downregulated (logFC < 0) subtracted from the number of assigned genes upregulated (logFC > 0) to the pathway divided by the square root of the number of genes assigned to the pathway). The bubble’s position on the plot corresponds to the pathway’s significance (the higher, the more significant). The size of each bubble represents the number of genes associated with the pathway. The bubbles are labeled with the pathway identifiers (IDs). A table displays the IDs, and the corresponding pathway description can be found on the right side.

For the WikiPathway 2021 Human database, Figure 7 shows that the enriched pathways with the expressed genes, which are nsp1 from SARS-CoV-2, inhibit translation initiation in the host cell, cell cycle, translation factors, and DNA repair pathways full network.

**Figure 8:**
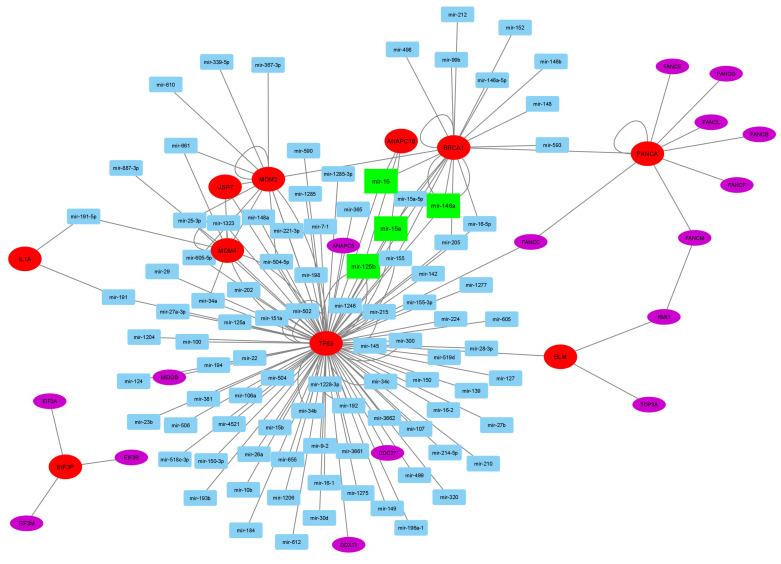
The melanoma’s gene–miRNA regulatory network is constructed from the TWAS-extended genes and the corresponding miRNAs. The large red ellipses represent the essential driver genes, and the large green rectangles denote the key driver miRNAs. The purple ellipses denote the genes, whereas the light blue rectangles represent the miRNAs.

For further analysis, the corresponding miRNA to the 76 associated genes were identified using Tfmir2, resulting in 106 miRNAs. From these genes and miRNAs, 26 genes and 96 miRNAs were involved in melanoma pathogenesis with 162 connections, as shown in Figure 8. In this gene–miRNA regulatory network, there are ten hotspot genes (*TP53* [10], *BRCA1*, *FANCA*, *BLM* [105], *USP7*, *MDM2*, *MDM4* [15], *IL1A* [16], *EIF3F* [122], and *ANAPC16*), whereas there are four hotspot miRNAs (mir-16 [123], mir-15a [124], mir-125b, and mir-146a [125]).

**Figure 9:**
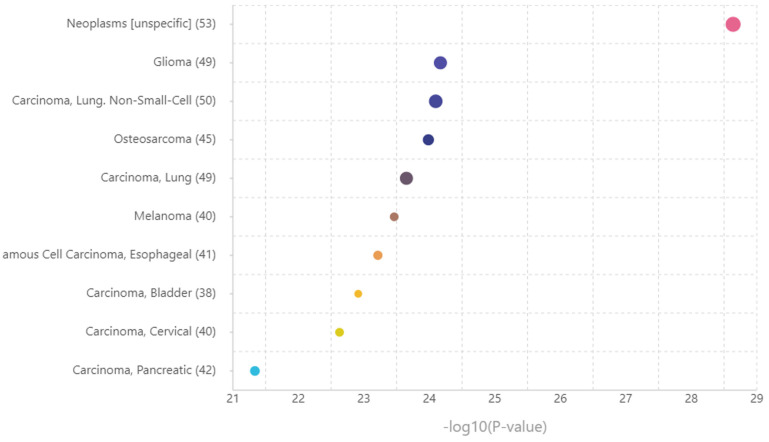
Top 10 diseases associated with the set of miRNAs related to the extended genes set. The X-axis shows −log10 (*p*-value), representing the strength of association between the disease and the miRNAs set. The Y-axis tabulates the top 10 associated diseases and the count of the miRNAs that play a role in the included disease. The size of the point correlates to the count of the miRNAs.

Consequently, a disease enrichment analysis was conducted using TAM 2.0 on the set of miRNAs identified by Tfmir2. Of the 106 miRNAs, 87 were provided in the disease enrichment analysis with 767 tested diseases. Figure 9 represents the top 10 associated diseases, with melanoma being the sixth disease (*p*-value = 3.44 × 10^−24^), confirming the proposed hypothesis.

The top three hotspot genes are *TP53*, *BRCA1*, and *MDM2*. The *TP53* gene, a key player in regulating the cell cycle, DNA repair, and apoptosis, is important in melanoma pathogenesis. While *TP53* mutations are rare, its function can be inactivated by overexpressed negative regulators like MDM2 and MDM4. This inactivation reduces TP53’s ability to prevent DNA damage caused by UV radiation, allowing melanoma to develop. Targeting the TP53 pathway, for example with MDM2 inhibitors, shows promise as a therapeutic strategy for melanoma treatment. Understanding TP53’s role in melanoma pathogenesis is crucial for developing effective treatments [126].

*BRCA1*, a gene linked to breast and ovarian cancers, also impacts melanoma. Germline and somatic mutations in *BRCA1* elevate melanoma risk and contribute to its progression. BRCA1 is crucial for DNA repair and interacts with the p53 pathway, influencing tumor development. Mutations also affect the tumor microenvironment and immune response, impacting melanoma progression and treatment response [127]. *MDM2* negatively regulates the p53 tumor suppressor pathway and influences melanoma development. It binds to p53, reducing its activity and promoting cell growth. Targeting MDM2 with inhibitors like Nutlin-3 can restore p53 function and induce tumor cell death, offering potential benefits in melanoma treatment [15,128,129].

The top four hotspot miRNAs are mir-15a, mir-16, mir-125b, and mir-146a. mir-15a is a crucial tumor suppressor in melanoma. It targets and reduces the expression of key genes involved in cell cycle progression and apoptosis, such as *CCNE1* and *BCL2*. Its reduced expression in melanoma leads to uncontrolled cell growth, enhanced cell survival, and tumor progression. Additionally, mir-15a suppresses multiple oncogenes and weakens the tumor-suppressive environment when its levels decrease [124].

mir-16 is a crucial miRNA in melanoma, regulating the cell cycle and apoptosis. Its reduced expression leads to uncontrolled cell proliferation and decreased apoptosis, promoting cell survival and resistance to apoptosis. Additionally, mir-16 directly targets oncogenes like *BMI1* and *EZH2*, promoting melanoma development and metastasis. Reduced mir-16 levels disrupt the balanced tumor-suppressive environment in melanoma cells, contributing to melanoma progression [123,130]. mir-125b has a critical role in melanoma by suppressing tumor growth and promoting cell death. It targets cancer-causing genes such as *BCL2*, *MCL1*, and *CDK6*, and its reduction leads to increased cell growth and resistance to cell death. Restoring mir-125b levels in melanoma cells shows promise as a treatment and combining it with other therapies could improve effectiveness [125,131].

The role of mir-146a in melanoma is complex, with both tumor-suppressing and tumor-promoting functions. It regulates inflammation and the immune response by targeting pro-inflammatory cytokines and molecules like IRAK1 and TRAF6, reducing inflammation and potentially affecting the tumor microenvironment. Additionally, mir-146a aids in immune evasion, enabling melanoma cells to evade immune surveillance. As a tumor suppressor, mir-146a targets genes involved in cell proliferation and survival, inhibiting oncogenes and promoting apoptosis. It also regulates the cell cycle; when downregulated, it can lead to uncontrolled cell proliferation and resistance to apoptosis. Furthermore, mir-146a inhibits metastasis by targeting genes involved in cell migration and invasion, such as *MMPs*, and influences the process of epithelial–mesenchymal transition (EMT), thereby impacting the metastatic behavior of melanoma cells [132,133].

## 5. Conclusions

Crucial associations with melanoma were discovered by applying TWAS on malignant melanoma of skin (C43) GWAS summary statistics (UK Biobank dataset) and TCGA-SKCM gene expression weights. In total, 26 genes showed a TWAS *p*-value lower than 0.05. Among them, five genes were not detected before for melanoma susceptibility, namely, *ANAPC13*, *RBAK*, *RPL36AP26*, *PROZ*, and *SPATA5L1*. To perform a functional pathway analysis and a disease enrichment analysis, the 26 genes were expanded by their physical and functional interacting proteins to end up with 76 genes.

The expanded gene set was responsible for regulating cell cycle phase transition and was associated with skin melanomas. In addition, the molecular interactions of the extended genes set were tested, identifying 106 miRNAs. The gene–miRNA regulatory network resulted in ten hotspot genes (*ANAPC16* was not detected before) and four hotspot miRNAs. Applying a disease enrichment analysis on the 106 miRNAs revealed that melanoma was included in the top associated diseases.

## Figures and Tables

**Table 1:** The TWAS-significant melanoma genes are arranged by genomic positions. Statistical significance was defined as TWAS.*p* < 0.05. Genomic positions were based on GRCh37/hg19 build. Previous studies linking the identified gene and melanoma were included. Other studies linking the gene with other diseases were presented.

Gene	CHR	Gene Start	Gene End	BEST.GWAS.ID	NSN	TWAS.*p*	Fold Change	Previously Studied in	Other Diseases Affected by the Gene
*AMIGO1*	1	110049447	110052336	rs12135954	46	0.00593	0.77	[36]	Autism [37]
*GSTM3*	1	110276554	110283660	rs3754446	51	0.01209	0.83	[38]	Autism [37]
*MDM4*	1	204485511	204677661	rs10458588	134	0.01635	0.33	[15,39,40,41]	Autism [37]
*COPA*	1	160258378	160313354	rs12140856	52	0.01683	2.42	[42]	Autoimmune interstitial lung disease-arthritis syndrome [43], acute respiratory failure [44]
*DENND4B*	1	153901977	153919154	rs11581644	37	0.04715	0.97	[45]	-
*RAB13*	1	153954128	153958806	rs12048137	30	0.04715	1.97	[17,46]	-
*IL1A*	2	113531493	113542971	rs4848300	51	0.0493	0.1	[16,47,48,49,50,51,52,53]	2q13q22.3 microduplication syndrome [54,55]
*ANAPC13*	3	134196547	134204865	rs9790169	38	0.0261	1.35	-	GATA2 deficiency with susceptibility to MDS/AMLDeafness-lymphedema-leukemia syndrome [56], mental retardation, autosomal dominant 47 [57]
*CRIPAK*	4	1385340	1389782	rs4974619	29	0.047	0.17	[58]	Mucopolysaccharidosis type 1 [59,60,61]
*LOC389458*	7	5023302	5112853	rs10234867	63	0.000236	1.2	-	Malignant tumor of prostate [62], Baraitser–Winter syndrome 1 [63,64,65]
*LOC441204*	7	26438339	26538594	rs1229677	138	0.015916	-	-	Alzheimer’s disease [66], gemcitabine chemoresistance [67]
*MTERFD1*	8	97251645	97273796	rs10092898	42	0.0183	2.35	[68,69]	Prostate adenocarcinoma [70], Furrow contractions [71]
*CBWD1*	9	121039	179075	rs636922	30	0.00317	0.6	[10,11]	Normal pregnancy [72]
*B3GAT1*	11	134248400	134281812	rs7123380	75	0.031	1	[73,74]	Schizophrenia [37], Paris–Trousseau thrombocytopenia [75]
*HOXC10*	12	54378946	54384060	rs4759316	56	0.00643	0.46	[76,77]	-
*DDX11*	12	31226779	31257725	rs2287465	26	0.04	0.78	[78]	Warsaw breakage syndrome [79,80,81,82,83,84,85]
*PROZ*	13	113812968	113826694	rs473598	38	0.0399	0.5	-	Factor X deficiency, factor VII deficiency [86], protein Z deficiency [87]
*DHRS1*	14	24759806	24769039	rs2180196	77	0.0111	0.32	[88]	-
*SPATA5L1*	15	45694519	45713614	rs8025019	33	0.039	0.54	-	Neurodevelopmental disorder with hearing loss and spasticity, deafness, autosomal recessive 119 [89]
*C16orf73*	16	1883984	1934295	rs12149777	91	0.000395	0.11	[90,91,92]	Spermatogenic failure 22 [93], tuberous sclerosis 2 [94,95,96,97], hemimegalencephaly [98]
*EIF3CL*	16	28699879	28747052	rs12448482	18	0.002754	2.12	[99]	Schizophrenia [37], hemimegalencephaly [98]
*FANCA*	16	89803959	89883065	rs258322	82	0.04941	2.66	[12,100,101,102,103,104,105]	Fanconi anemia [106], Fanconi anemia complementation group A [107], neuroblastoma [108]
*SCRN2*	17	45915049	45918699	rs17856536	53	0.033	0.81	[109,110,111]	-
*ALDH16A1*	19	49956473	49974304	rs11669675	45	0.0234	0.85	[112,113,114]	-
*UPK1A*	19	36157715	36169365	rs3761093	41	0.033	0.04	[13,115]	Dystonic disorder [75]
*EDEM2*	20	33703160	33865928	rs2425025	102	0.0421	2.27	[116,117]	Long QT syndrome [43]
*TEF*	22	41763392	41795328	rs2234059	36	0.00331	0.33	[118,119,120]	Immunodeficiency, common variable, 4 [121]

CHR: chromosome; BEST.GWAS.ID: rsID of the most significant GWAS SNP in locus; NSNP: number of SNPs in the locus; PTWAS: TWAS *p*-value.

**Table 2:** A list of the TWAS-significant genes extended to 76 genes using STRING. GeneCards was used to identify any alias of the gene name. The corresponding Ensembl IDs were included from GEPIA 2.

	Extended Genes	Aliases	Ensembl ID
1	*ALDH16A1*		ENSG00000161618.9
2	*ANAPC1*		ENSG00000153107.11
3	*ANAPC10*		ENSG00000164162.12
4	*ANAPC13*		ENSG00000129055.12
5	*ANAPC16*		ENSG00000166295.8
6	*ANAPC2*		ENSG00000176248.8
7	*ANAPC4*		ENSG00000053900.10
8	*ANAPC5*		ENSG00000089053.12
9	*ANAPC7*		ENSG00000196510.12
10	*APITD1*	*CENPS*	ENSG00000175279.21
11	*ARCN1*		ENSG00000095139.13
12	*B3GAT1*		ENSG00000109956.12
13	*BLM*		ENSG00000197299.10
14	*BRCA1*		ENSG00000012048.19
15	*C16orf73*	*MEIOB*	ENSG00000162039.14
16	*C17orf70*	*FAAP100*	ENSG00000185504.16
17	*C19orf40*	*FAAP24*	ENSG00000131944.9
18	*C1orf86*	*FAAP20*	ENSG00000162585.16
19	*CBWD1*		ENSG00000172785.18
20	*CDC16*		ENSG00000130177.14
21	*CDC23*		ENSG00000094880.10
22	*CDC26*		ENSG00000176386.8
23	*CDC27*		ENSG00000004897.11
24	*COPA*		ENSG00000122218.14
25	*COPB1*		ENSG00000129083.12
26	*COPB2*		ENSG00000184432.9
27	*COPE*		ENSG00000105669.12
28	*COPG1*		ENSG00000181789.14
29	*COPG2*		ENSG00000158623.14
30	*COPZ1*		ENSG00000111481.9
31	*CRIPAK*		ENSG00000179979.8
32	*DDX11*	*CHL1*	ENSG00000013573.16
33	*DENND4B*		ENSG00000198837.9
34	*DHRS1*		ENSG00000157379.13
35	*EDEM2*		ENSG00000088298.12
36	*EIF3A*		ENSG00000107581.12
37	*EIF3B*		ENSG00000106263.17
38	*EIF3CL*		ENSG00000205609.12
39	*EIF3D*		ENSG00000100353.17
40	*EIF3E*		ENSG00000104408.9
41	*EIF3F*		ENSG00000175390.12
42	*EIF3G*		ENSG00000130811.10
43	*EIF3H*		ENSG00000147677.10
44	*EIF3K*		ENSG00000178982.9
45	*EIF3L*		ENSG00000100129.17
46	*EIF3M*		ENSG00000149100.12
47	*FANCA*		ENSG00000187741.14
48	*FANCB*		ENSG00000181544.13
49	*FANCC*		ENSG00000158169.11
50	*FANCE*		ENSG00000112039.3
51	*FANCF*		ENSG00000183161.4
52	*FANCG*		ENSG00000221829.9
53	*FANCL*		ENSG00000115392.11
54	*FANCM*		ENSG00000187790.10
55	*GSTM3*		ENSG00000134202.10
56	*HOXC10*		ENSG00000180818.4
57	*IL1A*		ENSG00000115008.5
58	*IL1R1*		ENSG00000115594.11
59	*IL1R2*		ENSG00000115590.13
60	*LOC389458*	*RBAK*	ENSG00000146587.17
61	*LOC441204*	*RPL36AP26*	ENSG00000235828.5
62	*MDM2*		ENSG00000135679.21
63	*MDM4*		ENSG00000198625.12
64	*MTERFD1*	*MTERF3*	ENSG00000156469.8
65	*PROZ*		ENSG00000126231.13
66	*RAB13*		ENSG00000143545.8
67	*RMI1*		ENSG00000178966.15
68	*SCRN2*		ENSG00000141295.13
69	*SERPINA10*		ENSG00000140093.9
70	*SPATA5L1*		ENSG00000171763.17
71	*STRA13*	*BHLHE40* or *CENPX*	ENSG00000169689.14
72	*TEF*		ENSG00000167074.14
73	*TOP3A*		ENSG00000177302.14
74	*TP53*		ENSG00000141510.15
75	*UPK1A*		ENSG00000105668.7
76	*USP7*		ENSG00000187555.14

## Data Availability

The datasets supporting this article’s conclusions are available in the Gene Atlas database, http://geneatlas.roslin.ed.ac.uk/trait/?traits=60 (accessed on 16 July 2021), and on the FUSION website, http://gusevlab.org/projects/fusion/weights/TCGA-SKCM.TUMOR.tar.bz2 (accessed on 25 January 2022).
